# Particle focusing by 3D inertial microfluidics

**DOI:** 10.1038/micronano.2017.27

**Published:** 2017-07-31

**Authors:** Petra Paiè, Francesca Bragheri, Dino Di Carlo, Roberto Osellame

**Affiliations:** 1 Istituto di Fotonica e Nanotecnologie (IFN)-CNR and Dipartimento di Fisica-Politecnico di Milano, Piazza Leonardo da Vinci 32, 20133 Milan, Italy; 2 Department of Bioengineering, Department of Mechanical Engineering, California NanoSystems Institute, and Jonsson Comprehensive Cancer Center, University of California-Los Angeles, 420 Westwood Plaza, 5121 Engineering V, Los Angeles, CA 90095, USA

**Keywords:** 3D fluidic network, 3D particle focusing, Dean flow, inertial microfluidics

## Abstract

Three-dimensional (3D) particle focusing in microfluidics is a fundamental capability with a wide range of applications, such as on-chip flow cytometry, where high-throughput analysis at the single-cell level is performed. Currently, 3D focusing is achieved mainly in devices with complex layouts, additional sheath fluids, and complex pumping systems. In this work, we present a compact microfluidic device capable of 3D particle focusing at high flow rates and with a small footprint, without the requirement of external fields or lateral sheath flows, but using only a single-inlet, single-outlet microfluidic sequence of straight channels and tightly curving vertical loops. This device exploits inertial fluidic effects that occur in a laminar regime at sufficiently high flow rates, manipulating the particle positions by the combination of inertial lift forces and Dean drag forces. The device is fabricated by femtosecond laser irradiation followed by chemical etching, which is a simple two-step process enabling the creation of 3D microfluidic networks in fused silica glass substrates. The use of tightly curving three-dimensional microfluidic loops produces strong Dean drag forces along the whole loop but also induces an asymmetric Dean flow decay in the subsequent straight channel, thus producing rapid cross-sectional mixing flows that assist with 3D particle focusing. The use of out-of-plane loops favors a compact parallelization of multiple focusing channels, allowing one to process large amounts of samples. In addition, the low fluidic resistance of the channel network is compatible with vacuum driven flows. The resulting device is quite interesting for high-throughput on-chip flow cytometry.

## Introduction

On-chip flow cytometers promise several advantages in terms of cost reduction, portability, and increased device performance with respect to standard bench-top approaches. Indeed, the microfluidic approach favors analysis at the single-cell level, a reduced sample contamination, an increased analysis sensitivity and a higher processing automation^1,2^. To be truly competitive with standard instrumentation, these microfluidic devices should satisfy several challenging constraints: (i) a high throughput, necessary to process large amounts of samples in a reasonable amount of time; (ii) a low pressure drop over the microfluidic device, which basically means low fluidic resistance, thus allowing vacuum driven flows; (iii) optical accessibility inside the microchannel to allow high quality optical detection^3^. Moreover, a fundamental requirement is the capability to perform 3D particle focusing that forces the cells to flow one behind the other to enable enhanced optical alignment with better signal^4,5^. Unfortunately, due to the intrinsic 2D nature of the standard fabrication techniques, this achievement is difficult to obtain. So far, different methods have been proposed to obviate this limitation, but they usually envisage the use of external fields (such as acoustic^6^, electrical^7^ or optical fields^8^) or the presence of multi-lateral sheaths flows^9–12^. In recent years, inertial microfluidics has been successfully proposed to effectively manipulate particle positions in microfluidic channels^13–15^, by simply exploiting the channel geometry and the flow rate. This approach is extremely promising for flow cytometry applications, since it presents the great advantage of performing flow focusing within a single-inlet single-outlet microfluidic device without the need for external fields (that complicate the setups) and at high throughput. Indeed, for sufficiently high flow rates, inertial effects become relevant and generate forces on the flowing particles enabling their manipulation. Unfortunately, inertial lift forces in straight microchannels focus the particles in more than one equilibrium position, and require straight channels of several centimeters to be effective. An advantageous approach to reduce the number of equilibrium points and to speed up the particle repositioning is the introduction of curvature in the channel, which gives rise to a net secondary flow, known as Dean flow, that is orthogonal to the main flow direction. This introduces a drag force, proportional to the secondary flow velocity field, that facilitates the particle circulation in the channel cross-section, thus speeding up the particle repositioning to a subset of dynamic equilibrium positions^16,17^. Current microfabrication technologies limit inertial microfluidic devices to planar layouts, or two layer designs^18^. A typical microchannel geometry, used to generate a Dean flow, is the in-plane spiral^15,19,20^, which is characterized by a varying radius of curvature. For this geometrical layout the Dean flow effect is not constant in the device and is smaller given the large radius of curvature, thus long channels are typically required (in the order of few tens of centimeters^17,15^). Another planar approach uses asymmetric curving channels, made by a sequence of opposite curves alternating between tight and loose bending. In fact, in a planar geometry, a single-radius curvature would not be possible as it would provide a closed loop; the asymmetry in the curvature is introduced to generate a strong Dean flow in one curve and avoid counter effects in the subsequent curve^21^. However, since the reversing curves lead to counteracting flows, the compactness of this approach is still limited. In addition, this configuration only provides 2D focusing, leading to two focal positions in most channel geometries.

**Figure 1:**
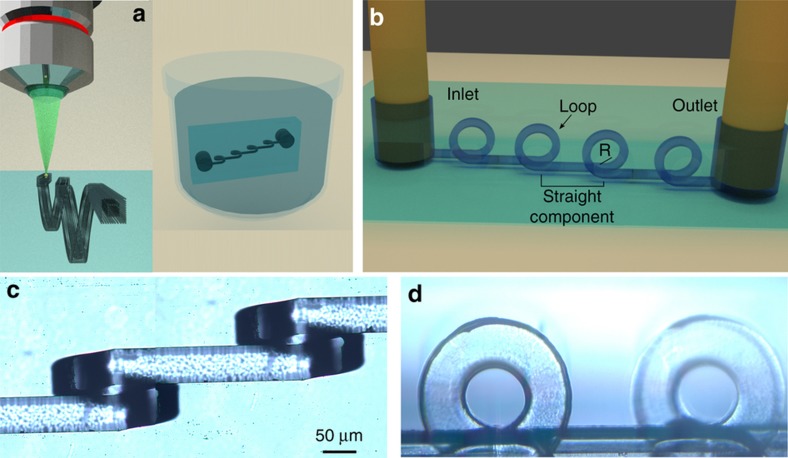
(**a**) Schematic of the fabrication process, first the device is irradiated by focused femtosecond laser pulses and subsequently exposed to an aqueous solution of hydrofluoric acid, allowing the three-dimensional (3D) microchannel formation. (**b**) 3D rendering of the proposed device, which is constituted by a sequence of straight channels intercalated by tightly curving loops. Microscope images of the fabricated device from (**c**) top and (**d**) side view. Panels **c** and **d** have the same scale bar.

In this work, thanks to the 3D capabilities of femtosecond laser micromachining followed by chemical etching (Figure 1a)^22,23^, we present an innovative 3D channel geometry that consists of the alternation of straight sections and vertical tightly curving loops (Figure 1b). Here exploiting the superposition of inertial and Dean effects, we obtain 3D particle focusing within a compact device, without the need for lateral sheaths flows. Moreover, we demonstrate compact channel parallelization with low fluidic resistance, yielding high throughput sample analysis, and making this device a valuable solution for sample focusing in on-chip flow cytometry.

## Materials and methods

### Fabrication

Femtosecond laser irradiation followed by chemical etching (FLICE)^22,23^ is a simple fabrication technique that allows one to fabricate 3D fluidic networks in fused silica glass substrates. It enables the realization of innovative microfluidic devices^9,24^ that take full advantage of the unique 3D capabilities of the technique. The fabrication consists of two distinct steps, schematically reported in Figure 1a. First, the glass substrate is irradiated using a focused femtosecond laser beam and then the substrate is exposed to an aqueous solution of hydrofluoric acid that will attack preferentially the irradiated areas, allowing the microchannel formation. The resolution of this fabrication technique is very high, indeed the volume modified by the focused femtosecond laser beam, limited only by diffraction, can be as small as a few hundreds of nanometers, which, after etching, could produce microchannels with a diameter of a few micrometers. In addition, it is possible to further improve the resolution by properly choosing the irradiation parameters and exploiting the self-assembled nanostructuring of the modified volume^25^, paving the way for nanofluidic networks. The accuracy of this fabrication method is also high, thanks in large part to the use of high precision motion stages for the sample translation and accurate control of the etching conditions. We typically have an accuracy in the channel layout of a few micrometers over millimeter-long microchannels. The main limitation of this technique, when fabricating directly buried structures, is the limited length of the microchannels that can be achieved (up to a few millimeters), due mainly to the difficulty in refreshing the exhausted acid inside long cavities. To avoid this limitation, in this work we have fabricated surface channels for the straight sections and have exploited the 3D capability of the technique only for producing the vertical loops. Therefore, the surface channels need to be sealed in a second step. We have irradiated the structures in a 20 mm×20 mm×1 mm fused silica substrate (Foctek Photonics, Fujian, China) using a commercial femtosecond laser system (femtoREGEN, HighQLaser, Rankweil, Austria), with an emission wavelength of 1040 nm and 1 MHz repetition rate. The second harmonic of this laser is focused through a ×63, 0.75 NA microscope objective in the fused silica substrate. The device is irradiated on the top surface of the glass, while access holes are irradiated in the glass volume until reaching the opposite surface to host external fluidic tubes. The sample is mounted on a translation stage (FIBERglide3D, Areotech, Pittsburgh, PA, USA) and it is translated with respect to the laser beam to obtain the 3D irradiation pattern that will define the microchannel shape; the sample scan speed (1 mm s^−1^) and the laser pulse energy (380 nJ) have been previously optimized to increase the etching selectivity and to reduce the irradiation step duration (the whole irradiation process is ~1 h). The etching step is performed by exposing the sample to a 20% aqueous solution of hydrofluoric acid (HF) at 35 °C. To facilitate the acid attack, the process is performed in a temperature controlled ultrasonic bath (B-3510, Branson, Danbury, CT, USA). After the etching exposure the sample is washed in a solution of deionized water and isopropyl alcohol. The etching process lasts ~2.5 h. The design, consisting of sequences of straight microchannels alternated with vertical loops (as reported in Figure 1b), is obtained by irradiating multiple parallel lines with 2 μm of reciprocal separation that define the external surface of the fluidic layout. The cross-sectional dimensions of the irradiated pattern are 40×40 μm^2^, resulting in a final microchannel with a 50×80 μm^2^ cross-section after the etching process. The enlarged dimensions are due to isotropic etching of fused silica, while the asymmetry is caused by the elliptical modification induced by the laser. To facilitate the removal of the material, we have irradiated a second structure inside the first structure with a reduced cross-section of 20×20 μm^2^ (as schematically shown in Figure 1a), thus helping the acid penetration and speeding up the etching process. In this work, we explored different geometrical parameters that characterize the device, such as the straight channel length (*s*), and the loop radius of curvature (*R*). Indeed, a helical microchannel (*s*=0 μm) and devices with longer *s* values such as 250, 600, and 1000 μm have been fabricated and compared in terms of focusing efficiency. Devices with a radius equal to 100 and 80 μm have also been fabricated and characterized. The loop step, i.e., the lateral separation between the loop input and output, is kept fixed at 120 μm. Before and after the whole loop sequence, a 1 mm long straight channel is fabricated, which allows us to separate the access holes from the characterization area, thus avoiding any background generated by the tubing fluorescence. We changed the number of loop-straight component iterations in the different devices according to the straight component length, and we decided to fix the input-output distance to approximately 10 mm, which means 30, 12, and 8 loops for *s* equal to 250, 650, and 1000 μm, respectively. The tubing access holes are obtained by etching an irradiation pattern composed of multiple coaxial helices that span from the channel surface to the opposite glass surface. The larger helix diameter is 700 μm, allowing a hole of approximately 800 μm after the etching that fits the PEEK tubing that we use for the fluidic connectorization. Figures 1c and d show the microscope images of a device after fabrication. In particular, Figure 1c reports the top view of a device characterized by a loop separation of 650 μm, while Figure 1d shows the corresponding side view, where it is possible to better observe the sequence of loops with a radius of curvature of 100 μm.

### Connections and sealing

The device is subsequently sealed with pressure-sensitive tape (Absolute QPCR seal), which affords good optical quality and fluorescence transmission. To increase the tape adhesion, the device is previously plasma treated using oxygen plasma (12 min at 0.4 mbar). External PEEK tubes (with outer and inner diameters equal to 0.78 and 0.5 μm, respectively) are subsequently inserted in the access holes previously fabricated and glued to the glass substrate with ultraviolet curable resin.

### Characterization setup and simulation

To test the device, a solution of fluorescent beads is inserted at a controlled flow rate and streak images of the fluorescence distribution are acquired to characterize the particle distribution in the microchannel cross-section. The experiments are performed under a fluorescence microscope in order to excite and collect the beads fluorescence, both from the top and from the side view of the device, depending on its orientation on the microscope. For this reason, both the top and lateral substrate surfaces are polished to ensure good imaging quality. The solutions are prepared by diluting polystyrene beads in deionized filtered water, and the experiments are performed using a concentration of approximately 2×10^6^ particles per mL. To test the device dependence on the particle size, two different samples are used with particle diameters of 15 and 7 μm, from Phosphorex (Hopkinton, MA, USA) and Sigma-Aldrich, St. Louis, MO, USA, respectively. The fluidic samples are delivered to the device using a high-pressure syringe pump, (KDS410, from KDScientific, Holliston, MA, USA), which permits fine control of the insertion flow rates. The fluorescence microscope is a LEICA DMI3000M and the camera is a LEICA DFC 310FX. Long-acquisition-time images are acquired during the experiments to retrieve the fluorescence streak lines, with an exposition time of approximately 1–2 s (averaging ~1 thousand particle trajectories). All the images are acquired after a few dozen seconds to wait for flow stabilization. The images are subsequently analyzed with custom Matlab (Natick, MA, USA) software to retrieve the vertical and horizontal particle distribution in the microchannel. Secondary flow velocity profiles are obtained using COMSOL Multiphysics (Burlington, MA, USA).

## Results and discussion

### Elements of inertial microfluidics

The design of our device is reported in Figure 1a, where it is possible to observe the sequence of loops and straight sections that compose the inertial microfluidic chip. A short digression on inertial microfluidics is useful to identify the forces responsible for particle movements and highlight the advantages of the present layout with respect to standard in-plane spirals. In a laminar regime, at sufficiently high Reynolds number (Re), inertial effects become relevant and give rise to a shear-gradient lift force due to the parabolic velocity profile that, combined with the wall-effect lift force, determine the location of particle equilibrium positions. These lift forces (*F*_L_) are channel geometry dependent and their efficiency in moving particles across the streamlines is influenced by the fluid flow rate and by the particle dimension. In rectangular shaped microchannels, if the channel width, *w*, is bigger than the height, *h*, (as it is in our geometry), the superposition of these forces leads to two distinct equilibrium positions, which are in the middle of the channel width, but close to the top and bottom surfaces^13^. On the other hand, the curvature of the channel introduces the secondary Dean flow, orthogonal to the main flow propagation direction and characterized by two counter propagating vortices^16^. The secondary flow intensity can be characterized by the Dean number, De=Re×(*D*_h_/(2*R*))^0.5^; where *D*_h_ indicates the microchannel hydraulic diameter, which is equal to 2*wh/(w+h)*, and *R* is the curvature radius. It follows that the tighter the curvature, the higher the Dean secondary flow intensity. This aspect is highlighted in Supplementary Figures S1a and b, where the secondary flow velocity fields of two loops, characterized by two different radii of curvature, are compared. The Dean flow introduces a net drag force (*F*_D_) on the particles orthogonal to the main flow direction that depends on the secondary flow velocity field^16^. If the Dean drag is much weaker than the lift forces, it does not affect the particle motion significantly, on the contrary if it is much stronger, then inertial lift forces are negligible and particles are trapped in the Dean vortices and rotate continuously. To determine the relative importance of the forces, an inertial force ratio has been introduced in the literature, defined as inertial lift over Dean drag:

Rf≈FL/FD=(2Ra2/Dh3)×f(Re,x/w,y/h,h/w),

where *a* is the particle diameter and *f*(Re, *x*/*w,y*/*h,h*/*w*) is a dimensionless function that depends on the channel Reynolds number, the particle location and on the channel aspect ratio. Considering a Reynolds number in the range of 20–95, the average *f* value has been shown to be approximately 0.02–0.03 (Ref. 26). In our devices, considering a radius of curvature of 100 μm, a hydraulic diameter of 62 and 15 μm diameter beads, we estimate a coefficient *R*_f_=0.005, which means that the Dean drag is predominant in the loop. With our layout, we can therefore decouple the Dean drag and the inertial lift forces, where the former are dominant in the loops to allow particles to sample the cross-section more efficiently, while the latter are dominant in the straight sections. We can thus separately control the two effects and find their optimal balance for the most effective particle focusing. In fact, lift forces define a limited number of equilibrium positions in the microchannel cross-section, while drag forces can move particles from one equilibrium position to another, and, if suitably engineered, enable 3D particle focusing in a single position.

### Operation principles

**Figure 2:**
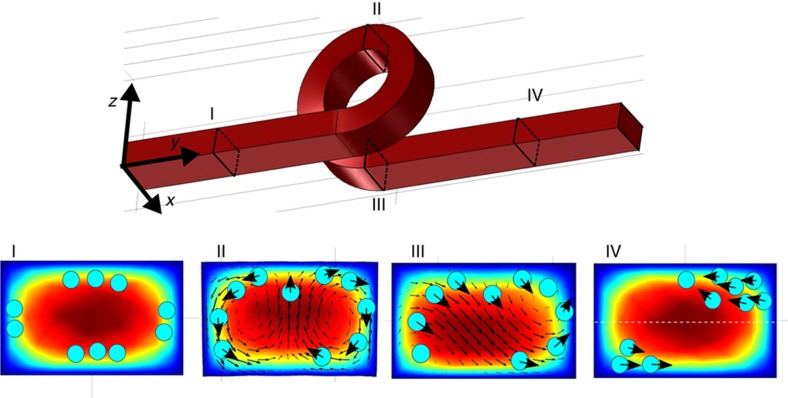
Schematic analysis of the inertial effects and particle motion in the different sections of the three-dimensional (3D) microfluidic channel. Comsol simulations of the velocity intensity and the secondary flow velocity field are reported. Panel I: particles begin to align close to the equilibrium position due to inertial lift forces, stable (close to the microchannel top and bottom) and unstable (close to the microchannel lateral surface) equilibrium positions are occupied. Panel II: particles rotate along the Dean vortices. Because the Dean drag is stronger in the channel center, particles are quickly removed from the center, favoring the formation of two lateral bands of particles. Panel III: an asymmetric Dean flow decay occurs, which favors the particle migration towards one corner of the channel. Panel IV: particles in the straight channel stop rotating and tend to align toward the equilibrium position of the rectangular channel. The iteration of this cycle, within the subsequent loops, allows the formation of a single focusing position in the top-right quadrant of the microchannel cross-section.

The device is based on the superposition of inertial lift forces and strong Dean drag. As previously discussed, it is possible to decouple the effects of the two forces in the straight section and the loop. In addition, a fundamental ingredient to obtain 3D particle focusing is the asymmetric Dean flow decay that occurs in our 3D layout. Indeed, after an in-plane channel curve, the Dean flow intensity decays in the subsequent straight section but maintains the same symmetry in the velocity field. In contrast, after a vertical loop, we must consider the effect of the channel translation along the *x* direction (e.g., helicity, Figure 2). This causes a break of symmetry in the Dean flow^27^, thus significantly altering the secondary flow velocity field (see the secondary flow decay comparison in Supplementary Figures S2a and b). This asymmetry can be exploited for 3D particle focusing, as schematically illustrated in the bottom panels of Figure 2. Panel I describes the particle behavior in the straight input channel, before the first loop. Here, inertial lift forces are the only forces acting on the particles and modifying their distribution. Hence the particles begin to align in correspondence with the two expected equilibrium positions (close to the top and bottom channel surface), but some particles still occupy unstable positions (close to the channel lateral surfaces). During the loop, Dean vortices occur, as shown in panel II. Here, the particles rotate along the vortices accordingly to the velocity vectors in terms of directions and intensity. For this reason, the main effect of this rotation is to empty the central area of the channel, where the Dean drag is stronger, thus favoring the formation of two lateral bands of particles. The flow rate optimization is fundamental in determining how much the particles move during the loop. Panel III shows the Comsol simulation of the secondary field decay just after the loop, where it is possible to observe the presence of two equilibrium positions, a stable (top-right) and an unstable position (bottom left). This velocity profile is responsible for the particles’ migration towards one corner of the microchannel cross-section, causing the single equilibrium position. The intensity of this velocity field rapidly decays, as reported in Supplementary Figure S2c, and it can be completely neglected after approximately 100–150 μm from the end of the loop. In the subsequent straight section, inertial lift is only acting on the particles. Hence the particles stop the circulation due to the Dean flow and move towards the equilibrium positions of the rectangular channel (panel IV). The next Dean flow will return the top-right particles to the corresponding corner, while it will move the bottom-left particles towards the center of the channel, where they will be dragged by the subsequent asymmetric flow decay to the top-right corner. The iteration of this cycle will empty the bottom-left equilibrium position favoring the one at the top-right, thus creating a single focusing position.

### Device characterization

**Figure 3:**
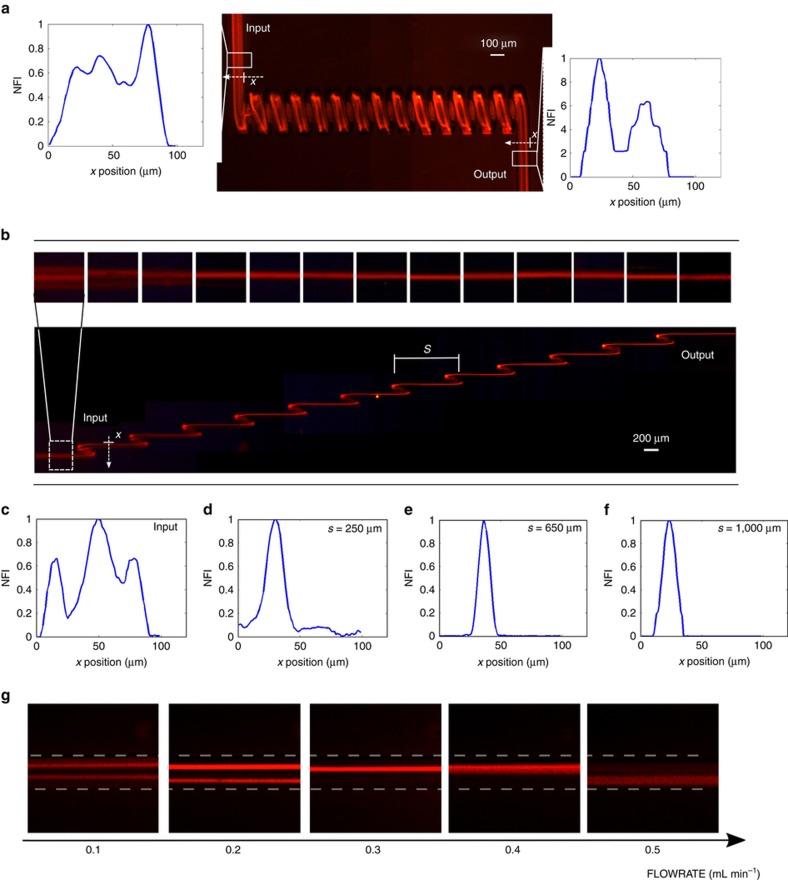
(**a**) Characterization of the fluorescence distribution generated by beads flowing in a microfluidic coil. The fluorescence distribution at the device input and output are analyzed and no focusing is obtained, as shown in the two insets that report the normalized fluorescence intensity (NFI) versus the channel width. (**b**) Characterization of a device with *s* equal to 650 μm, with top insets showing the magnified fluorescence streak lines in the straight sections after each loop. It is possible to observe that the particle focusing increases after each loop (narrowing of the streak line). (**c**) Fluorescence intensity profile at the device input. Fluorescence intensity profiles at the output of different devices with *s* values/number of loops equal to: (**d**) 250 μm/16; (**e**) 650 μm/12; (**f**) 1000 μm/7. An optimal focusing condition is found in configuration (**e**). (**g**) Fluorescence images at the end of device (**e**) versus flow rate. An optimal flow rate for efficient focusing is observed at approximately 0.3 mL min^−1^.

**Figure 4:**
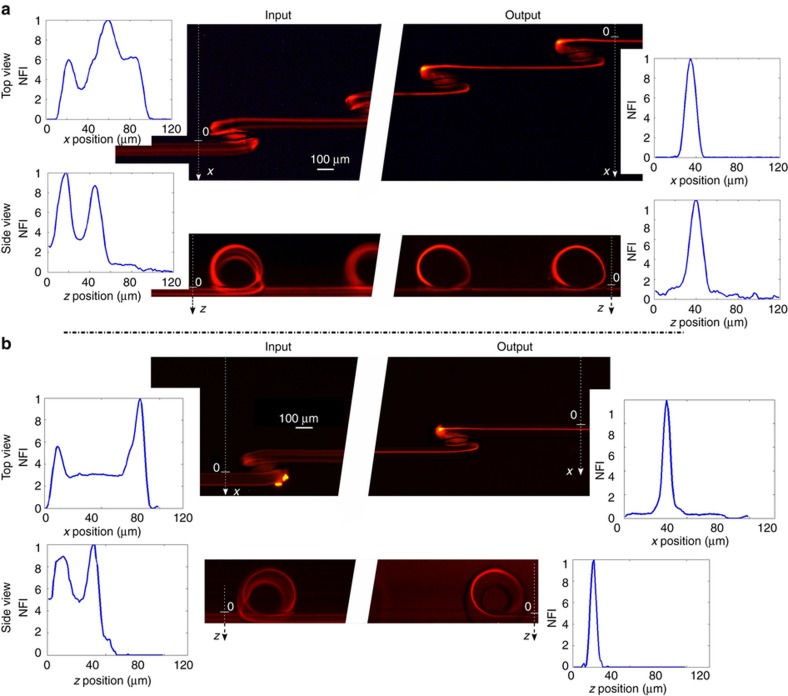
(**a**) Device validation with 15-μm diameter beads. Top and side views are reported, demonstrating the possibility of performing 3D particle focusing. The normalized fluorescence intensity (NFI) profiles at the input and output of the device are reported in the insets. (**b**) Same as (a) but with 6-μm diameter beads, thus highlighting the versatility of this focusing device with particles of different size.

We first investigated the role of the straight component length (the so-called *s* parameter). For this reason, we have fabricated and tested different devices characterized by different *s* values (0, 250, 650, and 1000 μm); the measurements are performed at a fixed flow rate equal to 0.3 mL min^−1^ and with a sample of 15 μm fluorescent beads. From this analysis, we found that a minimum *s* value is necessary to obtain focusing. Indeed, as reported in Figure 3a, the continuous coil (*s*=0 μm) is not effective in creating a single focusing position. It is worth noting that, before entering the microfluidic coil, particles are still widely distributed (in agreement with the particle distribution predicted in the model shown in Figure 2, panel I). However, at the output of the device we observe a distribution with two peaks, indicating that the main effect of the microfluidic coil is to empty the central area of the channel, thus favoring the distribution of the beads into two side bands. This effect is also observable by looking at the streak lines along the coil itself, and it is in agreement with a continuous Dean flow, as previously discussed. Figure 3b shows a top view of the device with *s* equal to 650 μm. Observing the fluorescence images after each loop, it is possible to note that the particle distribution narrows around a single focusing position. Figures 3c–f compare the device input with the best focusing obtained with *s* equal to 250, 650, and 1000 μm, after 16, 12, and 7 loops, respectively. Even though it is possible to obtain particle focusing with all three configurations, it should be noted that with the first configuration, the focusing is not complete, because there is still a fluorescence background signal around the peak. With higher *s* values, such as with the second and third configurations, the focusing quality is higher, and the particles are perfectly aligned. This analysis confirms the importance of the straight channel between the loops, where the asymmetric Dean flow decay and inertial lift forces stop the continuous particles circulation and confine them in just one position in the microchannel cross-section. To quantify the focusing efficiency, we analyzed the fluorescence streaks by measuring their full width at half maximum (FWHM). We observed FWHM values of 10 and 15 μm for the second and the third configuration, respectively. Because of the narrower particle distribution, we concluded that *s*=650 μm and 12 loops is the optimal configuration for a fixed footprint of ~10 mm, which corresponds to a microchannel length of 17 mm, when considering the rolled length of each loop. Supplementary Figure S3 shows the full device characterization. This length for focusing is relatively small compared to previous reports, which often require channels with lengths >30 mm. As previously discussed, the choice of the flow rate is fundamental to obtaining the correct particle focusing, since it influences the Dean drag and therefore the particles path along the secondary flow in the loop. The images in Figure 3g show the top view of the particle streak line acquired after 12 loops at different flow rates. It can be observed that there is an optimal focusing efficiency of ~0.3 mL min^−1^, while for the other flow rates the particle distribution is larger or has two peaks. For lower flow rates, the Dean decay is not strong enough to empty the unstable equilibrium position, thus explaining the double fluorescence streak line. At higher flow velocities, the strong Dean drag excessively mixes the particles in the loop, thus preventing the focusing in a single position. At 0.3 mL min^−1^, the device is characterized by a channel Reynolds number of 77 and by a Dean number equal to 43. With these flow rate conditions, we have characterized the 3D focusing capability of the device using 15 μm fluorescent polystyrene beads. The characterization is performed by analyzing the top and the side views of the particle distribution at the device input and at the output, as reported in Figure 4a. The corresponding image analysis reveals that, while the particles at the input are widely spread in the microchannel cross-section, at the output they are perfectly focused one behind the other both in the horizontal and in the vertical plane, allowing us to obtain the desired 3D particle focusing. By analyzing the FWHM distribution, we obtain an almost symmetric focusing: the peak is 10 μm large×12 μm high. The same characterization is performed also for particles with a smaller diameter, using 6 μm fluorescent polystyrene beads (Figure 4b). In this case, due to the dependence of inertial forces on the particle diameter, a new flow rate optimization was required. With an optimal flow rate equal to 0.2 mL min^−1^, we have been able to obtain a 3D focusing spot of 8 μm×7 μm (FWHM). The successful validation of the device with particles of very different sizes highlights the versatility of this approach, which gives rise to a microfluidic chip capable of working with a large variety of biological samples, simply by tailoring the proper flow rate value.

### Device optimization and multiple parallel focusing implementation

**Figure 5:**
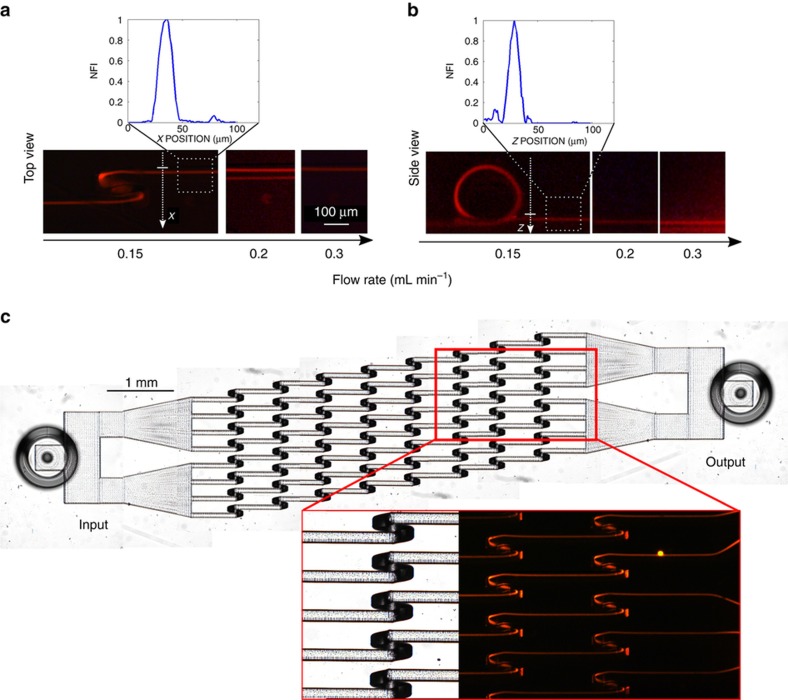
(**a** and **b**) show the characterization of the 80 μm loop-radius device performed with 15 μm beads at different flow rates. Top and side views are reported, showing an optimal flow rate at 0.15 mL min^−1^, which is different from the one obtained for the 100 μm loop-radius device. (**c**) Microscope image of the device with multiple parallel channels and the corresponding fluorescence image that demonstrates simultaneous particle focusing in all channels.

To further reduce the device length and therefore the fluidic resistance, we fabricated and characterized a device with a smaller loop radius of curvature, equal to 80 μm; the aim being to enhance the Dean drag intensity and therefore the secondary flow decay field, thus reducing the number of loops and the total length of the device required to perform the 3D focusing. The new device was tested with different flow rates using 15 μm polystyrene beads. The results, reported in Figure 5a and b, indicate the presence of an optimal flow rate value to obtain the 3D focusing, different from the one obtained with the previous device and equal to 0.15 mL min^−1^. In this case the number of loops required to perform the same level of focusing (the FWHM of the fluorescence distribution is equal to 10.5 μm) is indeed reduced with respect to the previous design and is equal to 8, giving rise to a footprint length of 7 mm corresponding to a microchannel length of 11 mm. In this case, it is also interesting to note that the number of loops can be further reduced and good particle focusing can be obtained after only 5 loops (FWHM equal to 12 μm), which corresponds to an 8.5 mm long microchannel (the complete device characterization is presented in Supplementary Figure S4). Moreover, thanks to the linear layout and the lack of lateral sheath flows, we have been able to parallelize the device, fabricating a single-inlet, single-outlet microfluidic chip that contains eight parallel microchannels. This geometry permits further enhancement of the device throughput, which now can process up to 1.2 mL per minute, without influencing the fluidic resistance. The microscope image of the new geometry is shown in Figure 5c, with the corresponding fluorescence characterization, highlighting the possibility to efficiently focus the sample simultaneously in all the parallel channels (see also the Supplementary Movie for the device validation). Due to the compactness of the optimized chip, the pressure drop required to process the sample at the correct flow rate for 3D focusing is far below 1 bar, that is, 0.65 bar. This pressure drop is compatible with a vacuum driven flow, which is ideal for flow cytometry applications.

## Conclusions

In this work, we demonstrated a compact, single-inlet/single-outlet microfluidic device, capable of performing 3D particle focusing for a wide range of particle dimensions. The chip is based on an innovative 3D layout and exploits inertial effects and asymmetric Dean flow in helical channels to manipulate the particle position at high flow rates. In addition, we have fabricated multiple parallel channels capable of performing 3D particle focusing simultaneously with a low pressure drop, thus increasing the sample-processing rate and making the chip suitable for high-throughput on-chip flow cytometry.
