# Supplementary material for: Particle focusing by 3D inertial microfluidics
Source: Microsyst Nanoeng. 2017 Jul 31;3:17027. doi: 10.1038/micronano.2017.27 (PMC6444990; doi:10.1038/micronano.2017.27)
Supplement: Supplementary Information [file micronano201727-s1.pdf]

Supplementary file

Particle focusing by 3D inertial microfluidics

Petra Paiè[1], Francesca Bragheri[1], Dino Di Carlo[2] and Roberto Osellame[1]

Microsystems & Nanoengineering (2017) **3,** 17027; doi:10.1038/micronano.2017.27; Published online: 31 July 2017

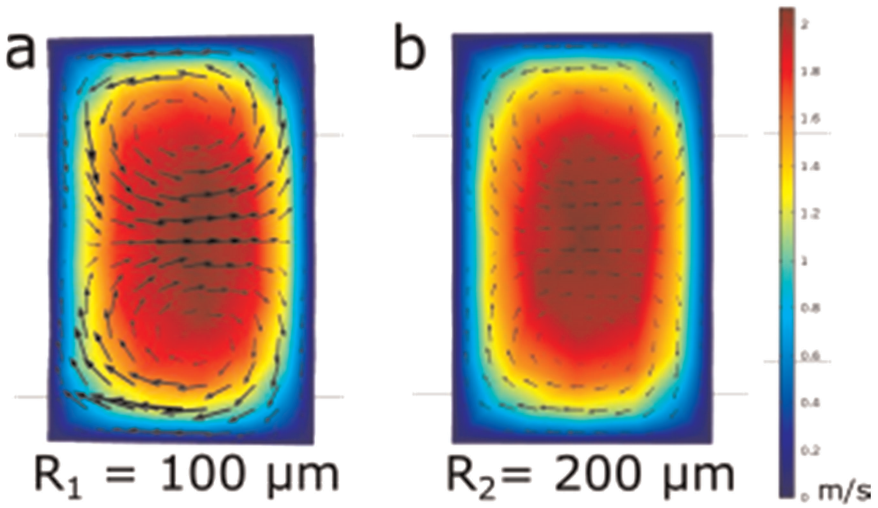

Figure S1 (**a**) and (**b**) show the different Dean flow occurring in loops with different radii of curvature, to observe how the larger the radius, the weaker the induced secondary flow is.

[1]Istituto di Fotonica e Nanotecnologie (IFN)-CNR and Dipartimento di Fisica-Politecnico di Milano, Piazza Leonardo da Vinci 32, 20133 Milan, Italy and [2]Department of Bioengineering, Department of Mechanical Engineering, California NanoSystems Institute, and Jonsson Comprehensive Cancer Center, University of California-Los Angeles, 420 Westwood Plaza, 5121 Engineering V, Los Angeles, CA 90095, USA
Correspondence: Petra Paiè (petra.paie@mail.polimi.it)

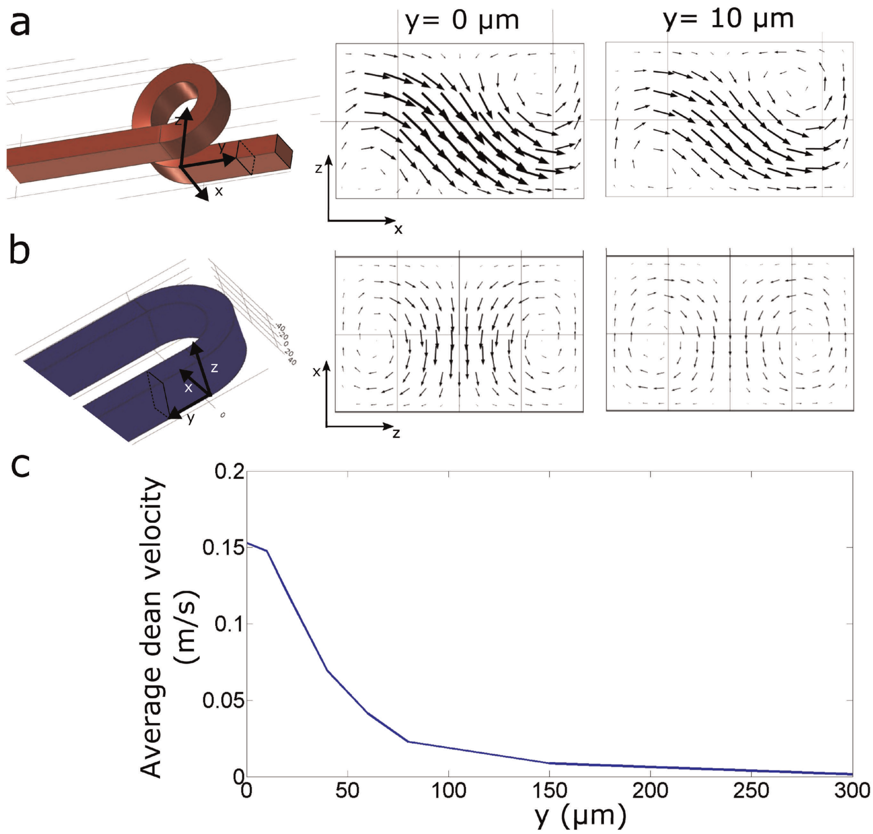

Figure S2 (**a**) Shows the asymmetric Dean decay profile that follows the vertical loop, (**b**) Shows the profile of the secondary flow after the in plane curvature, to note that the flow symmetry in this case is maintained. (**c**) Shows the secondary flow velocity (averaged on the channel cross section) as a function of the distance from the loop, which is obtained from Comsol simulation. The flow velocity halves after 40 µm and is completely negligible after 100–150 µm.

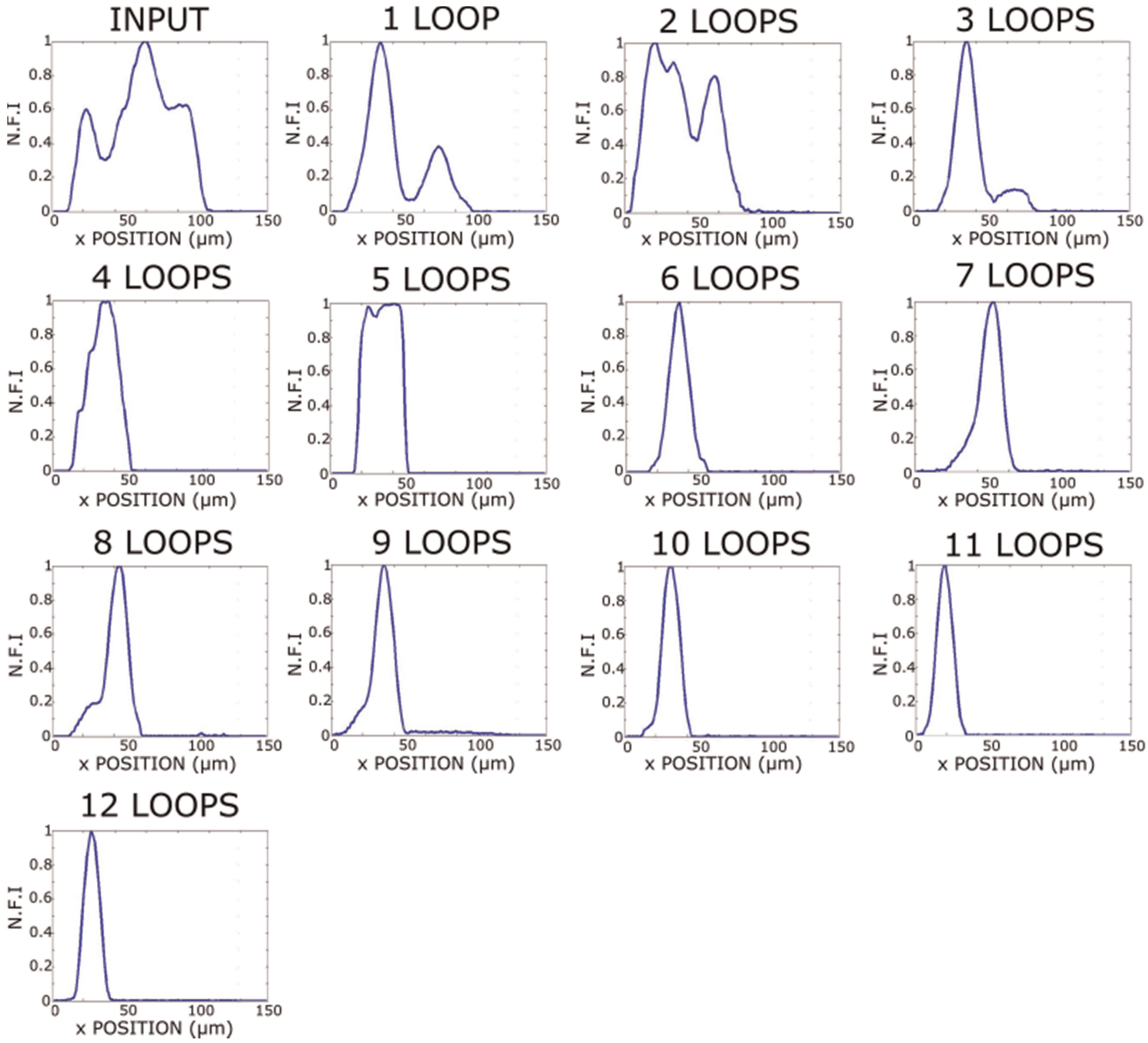

Figure S3 Analysis of the fluorescence streak line (Normalized Fluorescence Intensity, N.F.I) in the microchannel along the device with the straight component equal to 650 μm and the loop radius of curvature (R) equal to 100 μm. The device is analyzed after each loop, to retrieve the top view particle distribution.

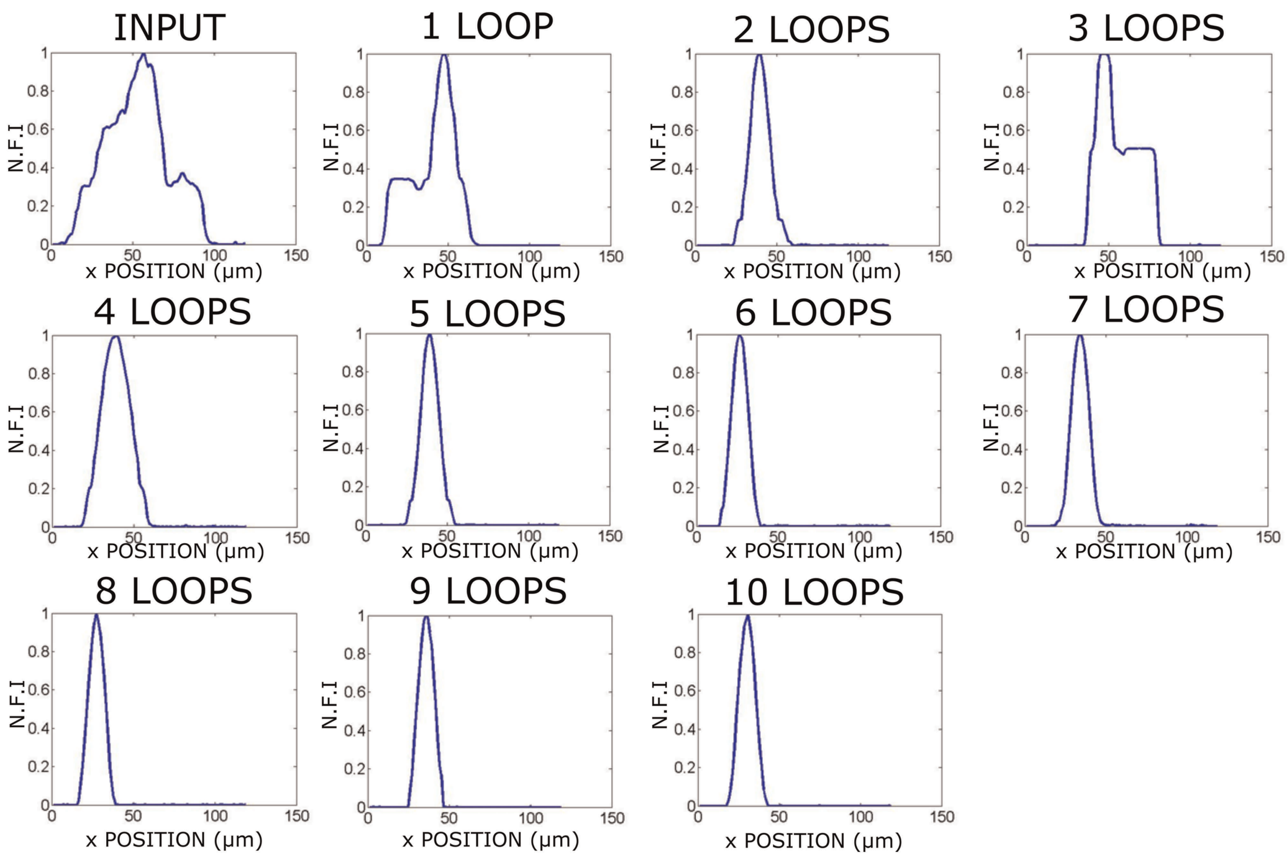

Figure S4 Analysis of the fluorescence streak lines along the device with the reduced loop radius of curvature (R = 80 μm). The device is analyzed after each loop, to retrieve the top view particle distribution.
